# Selection of controls

**DOI:** 10.1038/sj.bjc.6603975

**Published:** 2007-09-18

**Authors:** M von Euler-Chelpin, E Lynge

**Affiliations:** 1 Institute of Public Health, University of Copenhagen, Blegdamsvej 3, Copenhagen N, DK 2200, Denmark

**Sir,**

Dalton *et al* (2006) published a paper in the *British Journal of Cancer* on ‘The relation between socioeconomic and demographic factors and tumour stage in women diagnosed with breast cancer in Denmark, 1983–1999’.

Dalton *et al* (2006) conducted a case-control study using 23 808 women diagnosed with high-risk breast cancer as cases, and 6007 women diagnosed with low-risk breast cancer as controls. Using women with ‘basic school/high school’ education as baseline, they found the adjusted odds ratio for women with ‘vocational training’ to be 0.92, and that for women with ‘higher education’ to be 0.88.

In the discussion, Dalton *et al* (2006) claimed that their result ‘contrasts with the increasing risk for breast cancer with increasing education’ found by Danø *et al* (2004), and also based on Danish data. They commented that ‘It remains unclear whether the reason for the disparity by risk-group is delay in diagnosis or differing biology of cancers in the groups with less education and income compared with more advantaged groups’.

The explanation of the contrasting results is, however, much more simple. The study by Danø *et al* (2004) was a cohort study comparing breast cancer incidence rates and breast cancer mortality rates across educational groups. The Dalton *et al* (2006) study was, however, not a valid case–control study for estimating the relative risk of breast cancer incidence in high educated *vs* low educated women. Such a study would require that incidence density sampling had been used in the selection of controls, and Dalton *et al* (2006) did not follow this procedure. As it stands, the Dalton *et al* (2006) study simply presented odds of high-risk breast cancers versus low-risk breast cancers across educational groups.

The Dalton *et al* (2006) findings can as a matter of fact easily be derived from the Danø *et al* (2004) data. Using breast cancer mortality as a proxy for high-risk breast cancer, and breast cancer incidence as a proxy for low-risk breast cancer, the odds ratio pattern from the Danø *et al* (2004) data becomes almost identical to the odds ratio pattern from the Dalton *et al* (2006) data, Table 1.

This comparison of results from two Danish studies on socioeconomic risk factors for breast cancer really illustrates the importance of correct incidence density sampling of controls in case–control studies.

## Figures and Tables

**Table 1:** Rate ratios of breast cancer incidence and breast cancer mortality by educational group in Denmark 1970–1998, breast cancer mortality rate ratio divided by breast cancer incidence rate ratio (2), and odds ratios for high-risk *vs* low-risk breast cancer by occupational group in Denmark 1983–1999 (1)

	**Danø *et al* (2004)** **(2)**			**Dalton *et al* (2006)** **(1)**
**Education**	**BC incidence**	**BC mortality**	**BC mortality *vs* BC incidence**	**High-risk BC *vs* low-risk BC**
Low	1	1	1	1
Medium	1.21	1.07	0.88	0.92
High	1.38	1.19	0.86	0.88
